# Parallel fluorescence interrogation of multichannel chips for rapid point-of-care testing

**DOI:** 10.1007/s00216-026-06719-4

**Published:** 2026-08-08

**Authors:** Cosimo Trono, Simone Berneschi, Sara Tombelli, Ambra Giannetti, Barbara Adinolfi, Francesco Chiavaioli, Giampiero Porro, Luca Bolzoni, Giovanna Quarto, Romeo Bernini, Gianluca Persichetti, Genni Testa, Francesco Baldini

**Affiliations:** 1 Istituto di Fisica Applicata “Nello Carrara”, Consiglio Nazionale delle Ricerche, Via Madonna del Piano 10, 50019 Sesto Fiorentino, Italy; 2 Datamed Srl, Via Amerigo Vespucci, 2/4/6, Peschiera Borromeo, Milan, Italy; 3 Istituto per il Rilevamento Elettromagnetico dell’Ambiente, Consiglio Nazionale delle Ricerche, Via Diocleziano, 328, 80124 Naples, Italy

**Keywords:** POCT, Fluorescence, Diffractive optical element, Waveguide absorption filters, Multichannel microfluidic chip

## Abstract

The development of rapid and reliable point-of-care testing (POCT) devices requires optical platforms capable of combining fast readout, sensitivity, and robustness. In this work, a fully integrated optoelectronic platform for fluorescence detection in multichannel microfluidic chips is presented, based on a parallel optical interrogation strategy. The platform employs a diffractive optical element (DOE) to achieve simultaneous excitation of multiple microchannels and an array of waveguide absorption filters (WAFs) for parallel fluorescence collection and efficient rejection of excitation light. This configuration eliminates mechanical scanning and enables optical interrogation within a few seconds. The platform is integrated with a microfluidic handling system and dedicated control software, allowing automated execution of immunoassays. Stability and reproducibility were demonstrated through repeated chip loading experiments, showing low variability across microchannels. As a proof of concept, a fluorescence sandwich immunoassay for C-reactive protein (CRP) was implemented in plasma, achieving a limit of detection of 0.32 µg mL⁻^1^ with good intra- and inter-chip reproducibility. The proposed approach demonstrates the potential of parallel optical architectures for the development of rapid, scalable, and reliable fluorescence-based POCT platforms.

**Graphical abstract:**

**Figure:**
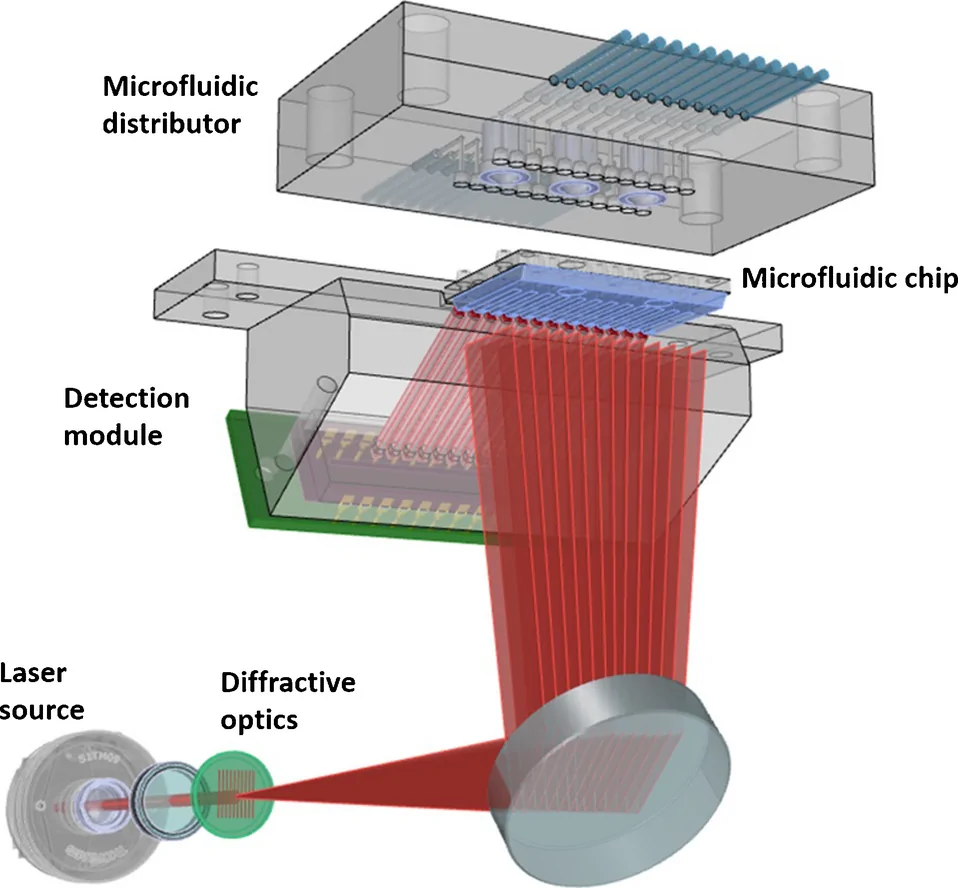

**Supplementary Information:**

The online version contains supplementary material available at https://doi.org/10.1007/s00216-026-06719-4.

## Introduction

In recent decades, optical chemo- and biosensors have undergone rapid development for the monitoring of a wide variety of samples, ranging from environmental to biomedical applications. These technologies provide valuable information on the quality and status of environmental matrices (e.g. soil, air, and water), as well as on human health [1–6]. Compared with other types of biosensors, optical biosensors exhibit a higher signal-to-noise ratio due to their complete immunity to electromagnetic interference from the external environment, as well as superior performance in terms of sensitivity and limits of detection [7–11]. Moreover, their small size and strong capability for integration with electronic and microfluidic components have enabled the recent development of portable instruments for in situ and real-time detection of pathogens or chemical species in environmental applications [12, 13].

The portability of optical biosensors has assumed a fundamental role in the biomedical field, leading to the development of the so-called point-of-care testing (POCT) devices, whose importance has steadily increased over the years [14–16]. These systems effectively address physicians’ need for rapid and reliable analytical tools, supporting timely diagnosis and informed therapeutic decision-making. POCT devices operate in a decentralised manner, close to the patient, thereby avoiding both delays associated with sample transport to centralised laboratories and waiting times for analytical results. This approach provides a twofold advantage: it represents a cost-effective solution while enabling the early establishment of a clinical pre-alert state, ultimately reducing the risk of adverse or fatal outcomes related to diagnostic and therapeutic delays. This aspect is particularly critical for time-dependent conditions such as sepsis, where even short delays in disease recognition or responsible pathogen individuation allowing the suitable treatment initiation significantly increase mortality in intensive care units, as well as for therapeutic drug monitoring of immunosuppressants in transplanted patients [17–20].

To date, the definition of a POCT device encompasses a broad range of systems, from benchtop instruments located at the patient bedside in hospitals, to compact analysers used in general practitioners’ offices, and up to fully portable or smart devices designed for home-care applications [21–23]. Moreover, many of these devices enable simultaneous multianalyte detection, allowing the measurement of panels of biomarkers through appropriate biochemical assays and thus providing rapid and specific responses for disease diagnosis and monitoring [14, 19, 23, 24].

In many cases, the core of a POCT device consists of dielectric materials, microfluidic channels, and optical components. These elements form the so-called multichannel optical chip, through which the biological sample is delivered in small volumes and optically processed to detect the presence of specific biomarkers, depending on the type of bioassay employed (e.g. sandwich, competitive, or binding inhibition assays) [25, 26]. In fluorescence-based detection strategies, the recognition elements used to identify these biomarkers are typically labelled with dyes, such as fluorophores or quantum dots (QDs) [17, 27–30]. Consequently, optimisation of the optical system for efficient excitation and collection of the emitted fluorescence signal becomes essential for the development of increasingly high-performance POCT devices, capable of meeting clinically relevant requirements in terms of detectable biomarker concentration ranges while also providing faster response times, thereby contributing to a reduction in patient length of stay [31].

In this context, the authors of the present work previously developed a POCT platform in which the multichannel optical chip was interrogated using a serial approach [27]. In this configuration, both the laser diode used to illuminate each individual chip microchannel and the optical fibre employed for lateral collection of the fluorescence signal at the chip output were translated step by step by means of a motorised stage. As a result, the system required several minutes to provide a meaningful analytical response, inevitably leading to increased waiting times. Such temporal constraints may be incompatible with the stringent requirements associated with the early diagnosis of certain pathologies, as discussed above.

To address these limitations and develop a POCT device capable of meeting such requirements, an alternative strategy is proposed herein. This approach relies on the parallel excitation and collection of the fluorescence signal within the same multichannel optical chip previously employed. To this end, a customised diffractive optical element (DOE) and a waveguide absorption filter (WAF) array were implemented to enable parallel channel excitation and simultaneous collection of the fluorescence signal at the chip output, respectively. Specifically, the DOE ensured uniform illumination of the individual chip microchannels by generating multiple light beams with transverse dimensions comparable to those of the channels themselves. Conversely, each WAF in the array effectively blocked the excitation radiation from the laser source while transmitting only the longer-wavelength fluorescence signal to the detection unit [32]. As a result, the proposed POCT platform exhibited extremely low response times, on the order of seconds. Parallel optical excitation or parallel detection strategies have been individually explored in the literature, for instance through wide-field, imaging-based multichannel fluorescence detection approaches and detector array readout schemes [14, 16, 24], or through multiplexed illumination, beam splitting, and beam shaping techniques enabling parallel excitation [1, 5, 6]. However, these approaches typically address only one side of the optical interrogation chain and generally preserve serial readout limitations, often requiring substantial modifications to the optical layout. For instance, imaging-based fluorescence systems, which rely on wide-field illumination and CCD/CMOS detection, enable parallel readout but require complex optical setups. This increases system size, cost, and alignment sensitivity, and can lead to a lower signal-to-noise ratio due to background autofluorescence and out-of-focus light. In contrast, non-imaging architectures based on spatially resolved excitation and guided fluorescence collection allow direct and efficient signal acquisition, with intrinsic background rejection and reduced system complexity. Building on this concept, the integration of parallel fluorescence excitation and collection systems into a fully static, compact optical architecture, specifically conceived to eliminate these constraints in the optical interrogation of microfluidic multichannel chips, may represent an original solution towards the development of next-generation POCT devices requiring rapid time-to-result and enhanced flexibility in multichannel analysis.

## Experimental

### The optical platform as a whole

The experimental prototype developed in this work is based on an integrated optoelectronic platform that combines optical excitation, fluorescence detection, and microfluidic sample handling within a single analytical architecture. The system was conceived to enable rapid and sensitive fluorescence immunoassays while maintaining compactness and scalability, key requirements for point-of-care testing (POCT) applications. At the core of the platform lies a custom polymer microfluidic chip, inspired in its geometry and operating principles by a previously reported design [27], and specifically engineered to interface efficiently with the optical interrogation system.

The microfluidic chip incorporates an array of parallel microchannels, allowing multiple fluorescence measurements to be performed simultaneously and thus paving the way for multiplexed analyte detection. While the chip architecture builds upon an established concept, the main innovation of the present prototype resides in the parallel optical interrogation strategy, encompassing both excitation and fluorescence collection, as well as in its seamless integration with the microfluidic component.

Optical excitation is achieved through a diffractive optics module capable of simultaneously and independently illuminating each microchannel, thereby providing spatially confined and uniform excitation over the sensing regions. The emitted fluorescence is spectrally filtered by an array of waveguide absorption filters (WAFs), each aligned with a corresponding microchannel, and subsequently detected by a single large-area detector, such as a CCD camera. This configuration enables efficient suppression of the excitation light and effective minimisation of background noise, while preserving a high collection efficiency for the fluorescence signal.

Although the platform is inherently designed for multianalyte fluorescence detection, the present study focuses on C‑reactive protein (CRP) as a model biomarker. This choice allowed validation of the proposed optical architecture and demonstration of the proof of concept of a benchtop POCT platform capable of delivering diagnostic results within a short time frame.

A detailed description of the optical design, optimisation of the individual subsystems, and final system integration is provided in the “Results and discussion” section.

### Materials and protocols

#### Reagents

Monoclonal antibodies and antigen for C-reactive protein (CRP) were obtained from commercial suppliers. The capture antibody was a mouse monoclonal anti-human CRP antibody (clone C5; Meridian Life Science, Memphis, TN, USA). The detection antibody was an Alexa Fluor 647 conjugated mouse monoclonal anti-human CRP antibody (clone C7; Novus Biologicals, Littleton, CO, USA). Human CRP antigen, supplied as a purified liquid preparation derived from human fluids, was purchased from Meridian Life Science (Memphis, TN, USA).

CRP-deficient human plasma was purchased from Innovative Research (Novi, MI, USA). According to the manufacturer, the CRP-deficient plasma exhibited an average CRP depletion of 99%, as determined by ELISA.

All the other reagents used for the preparation of phosphate‑buffered saline (PBS), for antibody immobilisation and for bioassay development were supplied by Merck (Milan, Italy).

#### Immobilisation of antibodies onto the microchannels of the chip

The CRP sandwich immunoassay was implemented on multichannel microfluidic chips following a two-step protocol comprising the binding of CRP to an immobilised capture antibody (clone C5), followed by the binding of the fluorescently labelled detection antibody (Alexa Fluor 647 labelled clone C7). Prior to assembly, the inner surface of the microchannels was functionalised by the manufacturer through deposition of a polymer layer providing amine (–NH₂) groups, enabling subsequent covalent immobilisation of antibodies. Covalent attachment of the capture antibody to the amino-coated microchannel surface was achieved via carbodiimide chemistry. Briefly, carboxyl groups on the capture antibody (500 µg/mL) were activated for 20 min using 1-ethyl-3-(3-dimethylaminopropyl) carbodiimide (EDC, 200 mM, 38.3 mg/mL) and N-hydroxysuccinimide (NHS, 50 mM, 5.8 mg/mL). The activated antibody solution was then injected into the microchannels and incubated for 1 h to allow covalent coupling to the surface amine groups. Following immobilisation, the microchannels were rinsed with PBS and subsequently blocked to minimise non-specific adsorption. Blocking was performed by injecting 1% (w/v) bovine serum albumin (BSA) for 30 min. Finally, extensive washing steps with PBST (PBS containing 0.05% Tween® 20) were carried out to remove unbound reagents.

#### Optical components and optoelectronic setup

The customised diffractive optical element (DOE) was fabricated on a bulk fused silica substrate by HOLOEYE Photonics AG (Berlin, Germany), according to specifications and optical design provided by the authors. The DOE micro-relief profile was realised by reactive ion etching, which transferred the phase pattern defined by a lithographic mask into the glass substrate, as detailed by the manufacturer (https://holoeye.com).

The waveguide absorption filters (WAFs) constituting the array were custom made by WTS Photonics CO., Ltd, (Fuzhou, China), by cutting and polishing a coloured glass filter (Schott RG645) originally provided as a 50 mm × 50 mm × 1 mm square. Each filter was processed to obtain a final geometry of 25 mm × 1 mm × 1 mm. As demonstrated in a previous study by the authors [32], WAFs with these dimensions are capable of fully suppressing the excitation signal while selectively transmitting the longer-wavelength fluorescence emission, yielding a signal-to-noise ratio (SNR) comparable to that achieved with high-performance interference filters. In addition, the high numerical aperture of the WAF enables improved fluorescence collection efficiency.

The optoelectronic excitation and detection system consisted of a laser diode (HL6322G, Hitachi Opnext) operating at 635 nm with a continuous‑wave output power of 15 mW, mounted on a temperature‑controlled laser diode mount (LDM21, Thorlabs) capable of stabilising the emission wavelength by maintaining the case temperature within ± 0.2 °C. To reduce the intrinsic beam divergence of the laser diode, an aspherical collimation lens (C110TME‑B, Thorlabs), featuring a focal length of 6.24 mm and a numerical aperture of 0.4, was employed. Simultaneous measurement of fluorescence signals was performed by a back‑thinned large‑area CCD linear image sensor: two versions of the camera were investigated, with a protective quartz window (Hamamatsu, model S11156-2048-01) and without it (Hamamatsu, model S11156-2048N-01). The detector presents an image size of 28.672 mm × 1.000 mm, a pixel size of 14 μm × 1000 µm, 2048 × 1 effective pixels, and a spectral response range from 200 to 1100 nm.

#### The microfluidic optical chip

The bioassay in the proposed point-of-care testing (POCT) device is implemented in a microfluidic optical chip, previously introduced by Giannetti et al. [27]. This architecture enables the acquisition of fluorescence signals with high sensitivity while maintaining compactness and robustness, which are essential requirements for POCT applications. The optical microfluidic chip, comprising 13 parallel microchannels (50 µm height, 600 µm width, 10 mm length), was manufactured by microfluidic ChipShop (Jena, Germany) according to specifications and design by the authors. The chips were fabricated from Topas cyclic olefin copolymer (COC), selected for its low autofluorescence at the excitation wavelength of 635 nm and its excellent optical transparency and bonding properties. Prior to assembly, the inner surfaces of the microchannels were functionalised by the manufacturer through deposition of a polymer layer providing amine (–NH₂) groups, enabling subsequent covalent immobilisation of antibodies. The geometry of the chip components was specifically designed for fluorescence-based bioassays, taking advantage of both the directional anisotropy of fluorescence emission from fluorophores located at sub- or near-wavelength distances from a dielectric interface and the efficient guiding of the emitted fluorescence through total internal reflection, optimising fluorescence signal collection efficiency while minimising background contributions [33].

## Results and discussion

### The optoelectronic excitation module

In the previous system [27], the microfluidic channel was illuminated from above along its entire length by means of a laser line generated by using a cylindrical lens. The resulting fluorescence, guided within the shaped upper part of the chip, was collected by a single plastic optical fibre and subsequently filtered by means of a high-pass interference filter and coupled to a photodetector. The interrogation of the 13 microfluidic channels was performed via sequential mechanical scanning. This procedure was characterised by a significant temporal duration (16 min), which considerably prolonged the overall assay time. Moreover, it introduced additional complexity in the device implementation, due to the requirement for a high-precision motorised translation stage capable of moving both the illumination module (laser source and cylindrical lens) and the detection module (optical fibre and photodetector) in a coordinated manner, in order to interrogate each of the 13 microchannels sequentially.

Both limitations can be effectively addressed through the use of the DOE, which enables the generation of a structured diffraction pattern composed of 13 distinct lines. This configuration allows the simultaneous interrogation of all 13 microchannels of the chip. This parallel excitation approach eliminates the need for mechanical scanning, thereby significantly reducing the total acquisition time and improving temporal resolution.

The customised DOE was designed with the following specifications:

- material: glass
- DOE dimensions (diameter × thickness): 8 mm × 1.2 mm
- geometry of the diffraction pattern: parallel lines
- number of lines: 13
- design wavelength: 635 nm
- inter-line spacing (at 15 cm): 2 mm
- line length: 10 mm
- pattern total size (at 15 cm): 24 mm × 10 mm

**Fig. 1:**
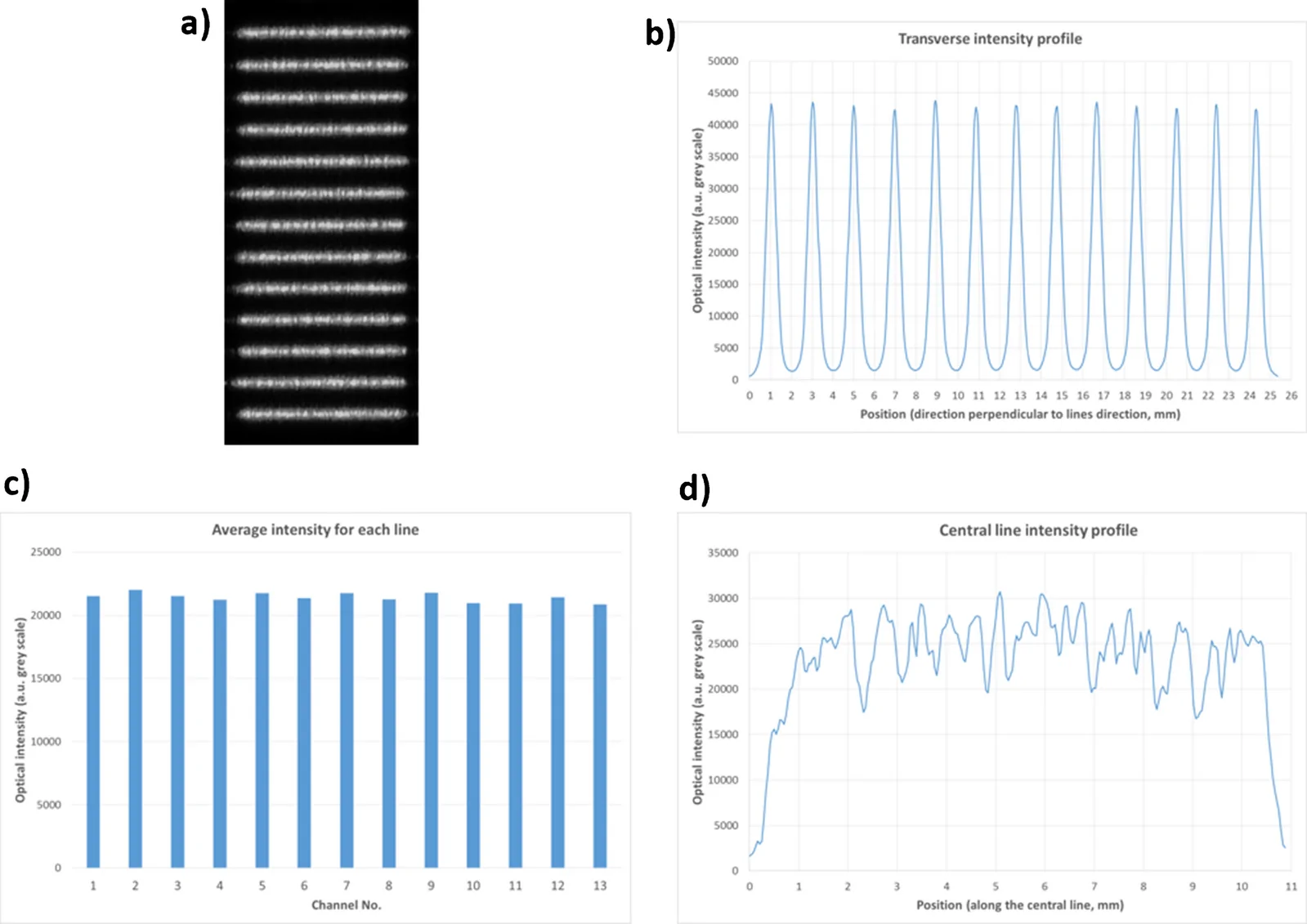
Characterisation of the diffraction pattern generated by the diffractive optical element (DOE): **a** photograph of the pattern projected onto a screen, showing the 13 parallel excitation lines; **b** transverse intensity profile measured at the centre of the pattern; **c** average optical power distribution across the 13 lines; and **d** longitudinal intensity profile of the central line

The use of a laser diode as an optical source led to the optimization of the diffracted pattern facilitating the system integration. Due to the intrinsic beam divergence of the laser diode, the emitted beam exhibited an elliptical profile. Partial collimation was achieved using the aspherical lens. This configuration reduced, without completely eliminating, beam divergence, resulting in an elliptical spot on the DOE with approximate dimensions of 1 mm (major axis) and 0.3 mm (minor axis). The LD was aligned with the DOE such that the major axis of the elliptical spot was parallel to the orientation of the diffracted lines. This alignment condition maximised the overlap between the excitation pattern and the microfluidic channel geometry, ensuring a more uniform distribution of optical power across all channels. Figure 1 shows the diffracted pattern projected onto a screen (a), as well as the transverse intensity profile at the centre of the line pattern (b), the average intensity distribution for all 13 lines (c), and the intensity profile of the central line (d). Figures S1 and S2 in the Supplementary Materials show the diffracted pattern, the intensity profile of the central line, the average intensity distribution, and the transverse intensity profile, both for the case in which the major axis of the elliptical LD spot is oriented transversely to the lines and for the case of a perfectly circular spot. It is worth noting that the typical anisotropic divergence of the laser diode, in this case, enables an extremely uniform optical power distribution across the 13 lines and, consequently, across the 13 channels of the microfluidic chip. This uniformity is critical to minimise channel-to-channel variability in fluorescence excitation, directly contributing to improved quantitative accuracy in multichannel measurements. The longitudinal intensity fluctuations observed in Fig. 1d originate from laser speckle, resulting from coherent illumination combined with phase modulation introduced by the DOE. While individual lines exhibit local variations (relative standard deviation around 12%), these fluctuations are largely uncorrelated and therefore significantly reduced when considering the total illumination. The integrated intensity over all 13 lines shows a much lower relative standard deviation of 1.7%, indicating stable overall optical power delivery despite local inhomogeneities. The influence of the illumination conditions was further assessed by comparing different beam profiles at the DOE plane. A perfectly collimated laser beam producing a circular spot (Figure S2 in the Supplementary Materials) leads to a more irregular intensity distribution (relative standard deviation of 54%), with fluctuations propagating to the integrated signal (10% standard deviation), due to inefficient speckle averaging. To mitigate this effect, a slightly non-collimated diode laser beam with an elliptical spot profile was employed. This reduces the effective spatial coherence at the DOE and promotes partial speckle averaging, improving illumination uniformity and reducing integrated intensity fluctuations from 10 to 1.7%.

**Fig. 2:**
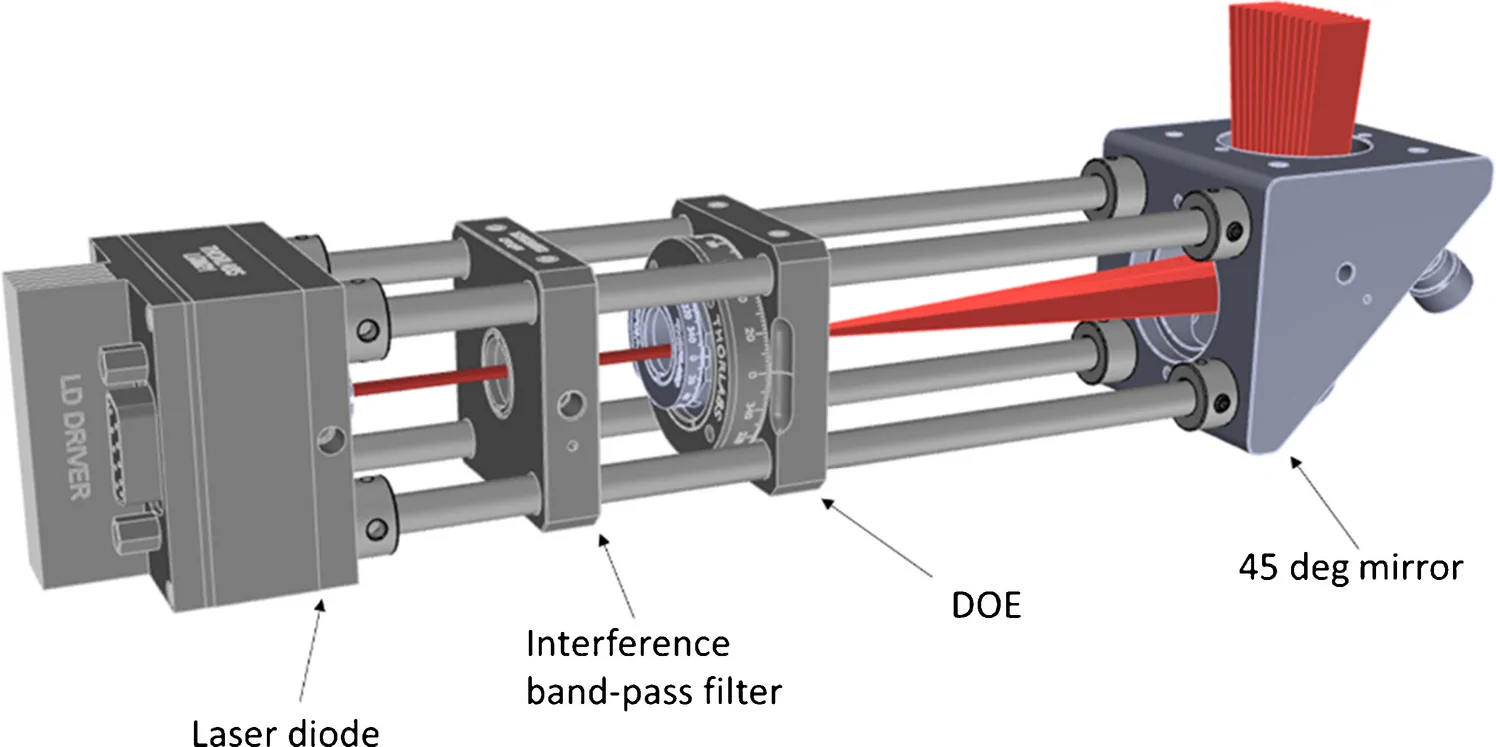
Rendering of the fluorescence excitation module. The setup includes the laser diode, bandpass interference filter, diffractive optical element (DOE), and 45° mirror used to direct the structured beam onto the microfluidic chip

The rendering of the fluorescence excitation setup is shown in Fig. 2, highlighting the laser source, the bandpass interference filter, the DOE, and the 45° mirror used for chip illumination. The compact and fully static optical configuration enhances system robustness and eliminates alignment drifts associated with moving components.

The resulting module was then integrated into a light-shielded section of the final prototype, described in the following sections.

### The optoelectronic detection module

To maintain the parallelism of the optical interrogation system and allow the possibility of rapid optical measurements, the multiplexing feature of the excitation system must necessarily be replicated on the signal collection one. This design ensures that the benefits of parallel excitation are preserved along the entire optical interrogation chain, avoiding bottlenecks in signal acquisition.

**Fig. 3:**
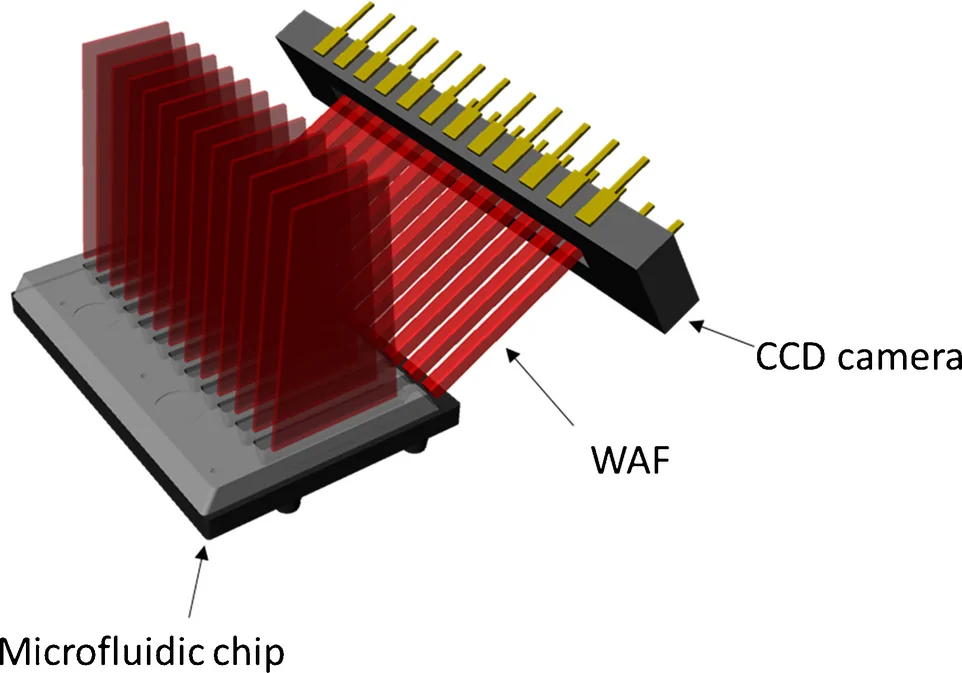
Rendering of the fluorescence detection module. The waveguide absorption filter (WAF) array is aligned with the microfluidic chip output and coupled to a linear CCD detector. The system geometry, including the tilt of the WAF array with respect to the chip surface, is optimised to maximise fluorescence collection efficiency while minimising background contributions

To achieve this, the WAF array had to be appropriately interfaced on one side with the microfluidic optical chip and on the other with the large-area CCD linear sensor. On the chip side, the end faces of its top part, which acts as a waveguide for the fluorescence signal propagating within it through successive total internal reflections, were angled at approximately 60°. Thus, most of the guided fluorescence signal resulted perpendicular to the sloped surface, passing through it [34]. As a result, the WAF array was appropriately tilted to achieve optimal facing and, consequently, maximise the fluorescence signal collection (Fig. 3). This geometrical optimisation maximises the coupling of the guided fluorescence into the WAFs, thereby improving signal collection efficiency.

**Fig. 4:**
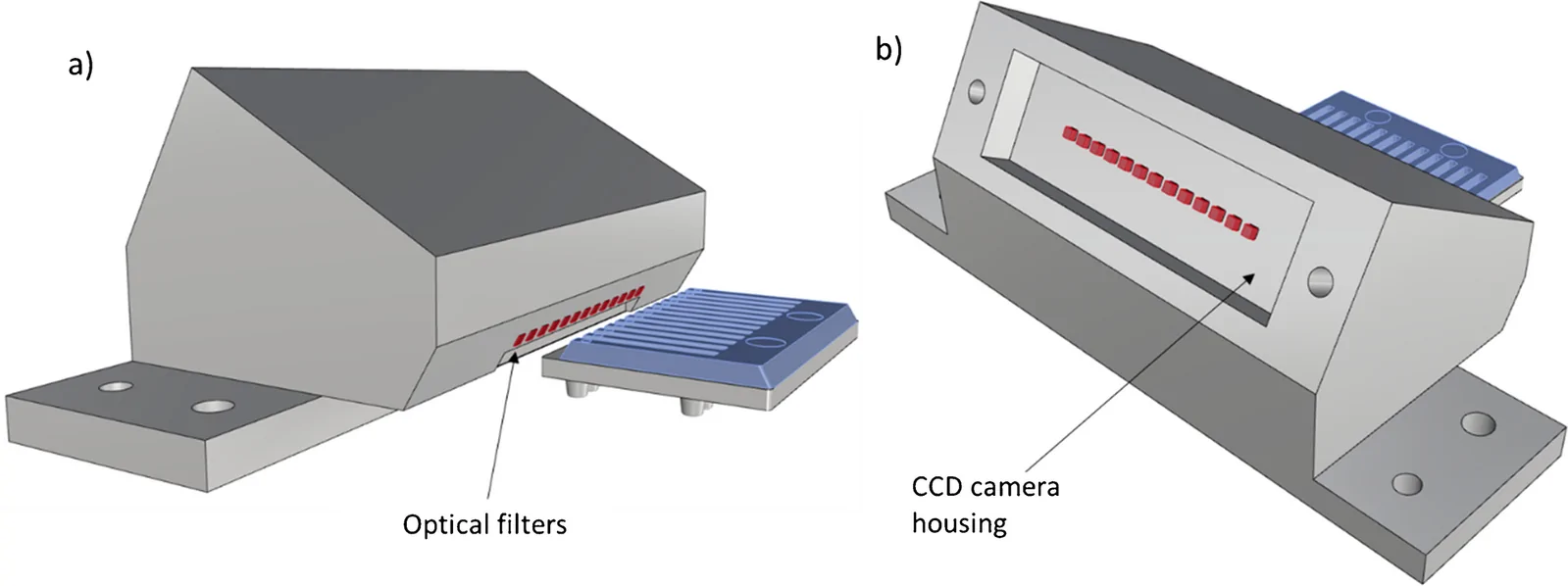
Custom-designed light-shielding holder for the WAF array, realised by 3D printing: **a** front view showing the alignment of the 13 angled channels with the microfluidic chip output; **b** rear view showing the housing for the CCD camera

In addition, it was necessary to prevent even the smallest scattered light component (i.e. diffused stray light) from the surrounding environment from reaching the WAFs transversely, thus influencing the measurement based on the fluorescence signal. For this purpose, a special holder in black polymer (Vero® black resin) was realised, under the design of the authors, by the company Topp SRL (Sandrigo (VI), Italy) with selective laser sintering (SLS) 3D printing technique. The shielding block featured 13 square-section through-holes, each about the size of a WAF, angled to maximise the collection of the fluorescence signal from the chip microchannels. On the front side, the block featured a specially shaped cross-section, with the 13 holes spaced equal to the angled faces of the chip cover. This ensured proper interfacing between the two parts (Fig. 4a). Additionally, a suitable housing for the CCD camera was created on the rear of the block (Fig. 4b). The large-scale photodetector was then secured to the block with screws. The WAFs were manually inserted into each hole, so that their front face was slightly inside the hole and, at the same time, their rear face was as close as possible to the photosensitive surface of the detector. The use of a dedicated light-shielding structure significantly reduced the contribution of stray light, leading to an improved signal-to-noise ratio in fluorescence measurements.

**Fig. 5:**
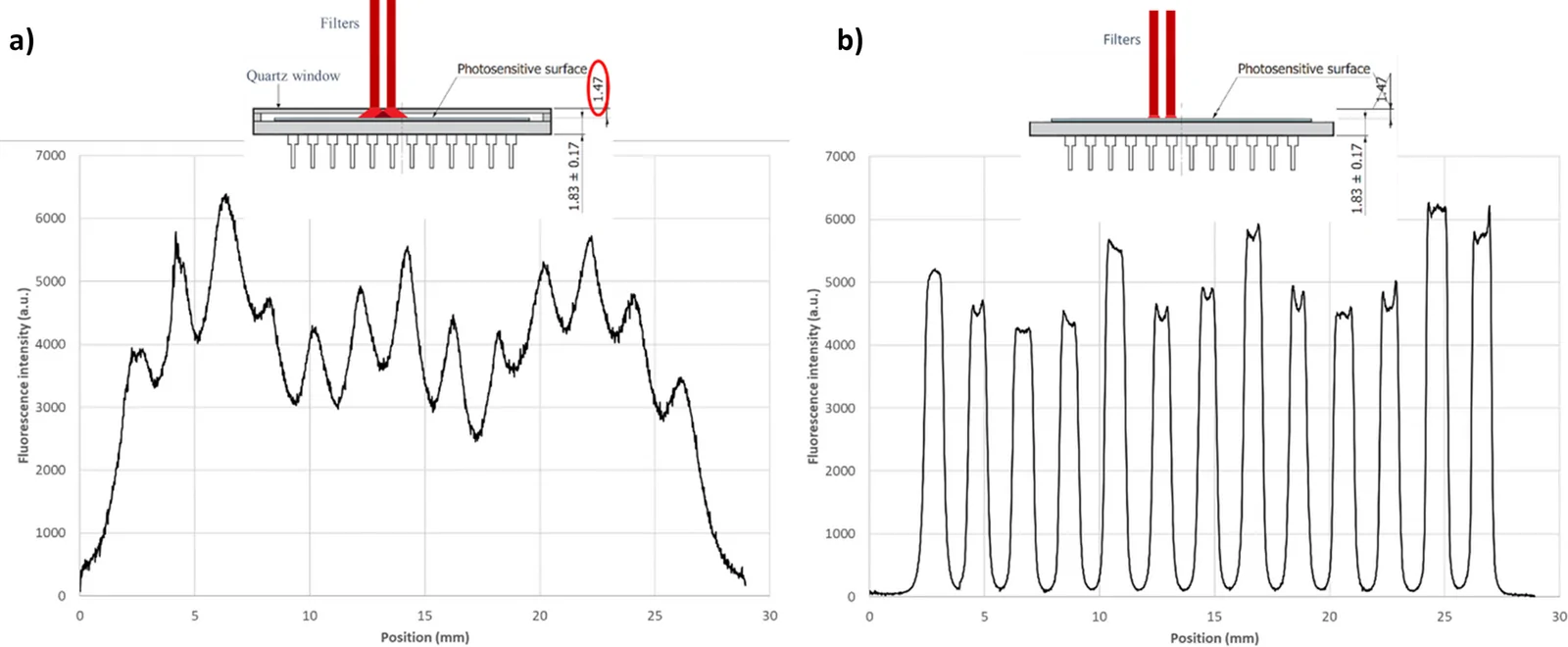
Comparison of fluorescence signal acquisition using two CCD configurations: **a** standard camera with protective quartz window (S11156-2048-01 CCD camera), resulting in increased optical spread and potential inter-channel overlap; **b** window-less camera (S11156-2048N-01 CCD camera) enabling quasi-contact coupling between the WAF output and the detector surface, thereby reducing crosstalk and improving spatial resolution of the multichannel readout

On the CCD camera side, the first tested version was the conventional one (S11156-2048-01), featuring a quartz window that protects the sensitive area. This configuration implies a minimum distance of 1.47 mm between the photosensitive surface and the end (i.e. the output face) of each WAF, causing the different fluorescence signal contributions to spread and potentially overlap near the detector surface (Fig. 5a). In the second custom-made version of the CCD camera (S11156-2048N-01), the absence of the quartz window allowed the WAFs to be brought as close as possible—in a quasi-contact configuration—to the sensitive surface of the photodetector, drastically reducing the possible optical crosstalk (Fig. 5b). Reducing the distance between the WAF output and the detector surface limits the spatial spread of the fluorescence signal, thereby mitigating inter-channel overlap and preserving the spatial resolution of the multichannel readout. This configuration is essential to ensure accurate signal discrimination between adjacent channels, which is a key requirement for reliable multiplexed detection.

Although all necessary countermeasures were taken to address unwanted phenomena (e.g. stray light; crosstalk during signal collection), inter-channel optical crosstalk occurring between adjacent channels within the microfluidic chip was still present. This issue arose from the close spacing of the channels on the chip (approximately 2 mm apart, along of more than 10 mm) which allowed fluorescence travelling along a specific channel to couple into neighbouring channels through scattering and multiple reflections. The parallel interrogation system approach makes it necessary to implement alternative strategies aimed at reducing the possible presence of inter-channel crosstalk. This issue was addressed through post-processing of the microfluidic chip by filling the spaces between the channels with commercial black silicone. The effectiveness of this approach was assessed by filling all the channels with PBS and then sequentially replacing the PBS with a fluorescent solution (IgG labelled with Alexa Fluor 647 5 µg/mL), acquiring the optical signal after each step. Crosstalk was evaluated by comparing the signal collected from a given channel filled with PBS before and after the sequential filling of the other channels with the fluorescent solution. To specifically evaluate the effect of the black silicone, the spaces between channels 5, 6, 7, 8, 9, and 10 were filled with this material. The improvement achieved with the use of black silicone is evident, as reported in Table 1, where thicker black vertical lines indicate the presence of the black material in the inter-channel spaces. Each row of the table provides the signal variation observed in channels filled with PBS when the *i*-th channel is filled with the fluorescent solution. For instance, filling channel 2 produces a 17.34% signal variation in channel 3, a 5.13% variation in channel 4, and so on. In contrast, filling channel 6—shielded from channel 5 by black silicone—results in only a 0.33% variation in channel 7 and negligible effects in the remaining channels. Minimising inter-channel crosstalk is essential to preserve the analytical specificity of each sensing channel and to prevent false signal contributions in multiplexed assays.

**Table 1.**
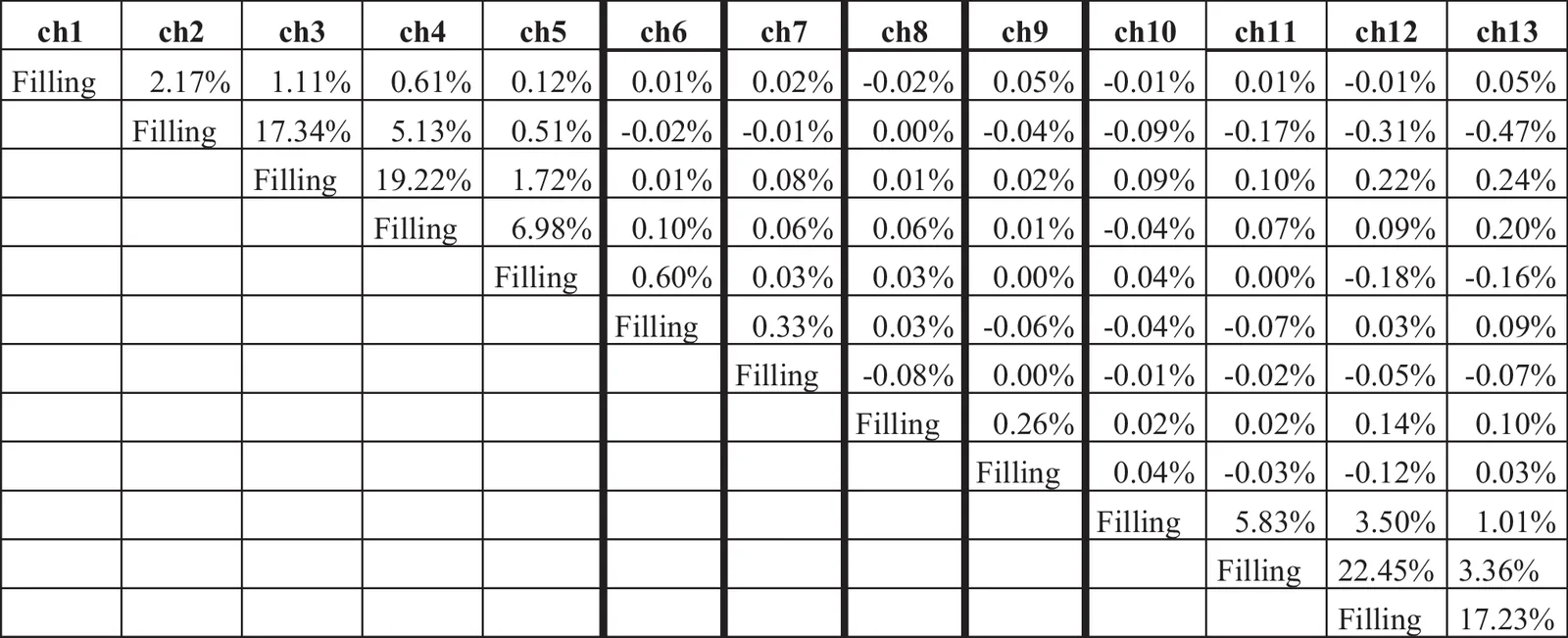
Relative signal variation in adjacent channels induced by filling a specific channel with the fluorescent solution. Each row reports the signal variation measured in channels filled with PBS when the *i*-th channel is filled with the fluorescent solution. The thicker black vertical lines indicate the presence of black silicone in the interspace among adjacent channels from channel 5 to channel 10

**Fig. 6:**
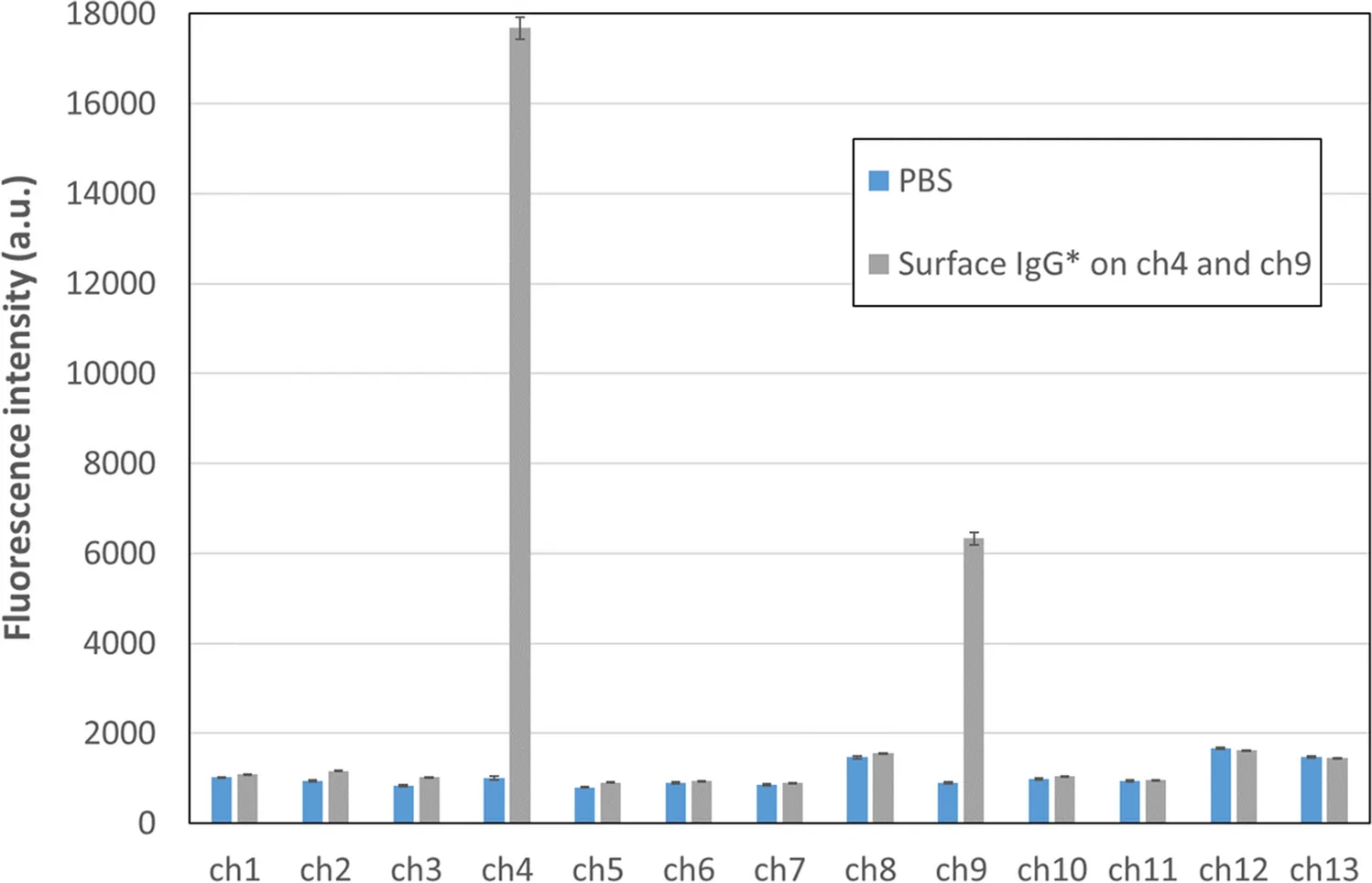
Evaluation of inter-channel optical crosstalk under conditions mimicking a heterogeneous assay. The histogram shows the fluorescence signal measured in all channels filled with PBS before and after the selective immobilisation of labelled antibodies (IgG labelled with Alexa Fluor 647 5 µg/mL on channel 4 and 2 µg/mL on channel 9) on channels 4 and 9

The effectiveness of the approach was further assessed in a context closer to the heterogeneous assays by immobilising IgG labelled with Alexa Fluor 647 5 µg/mL and 2 µg/mL (IgG* in Fig. 6) directly only on channels 4 and 9, respectively. Figure 6 shows the histogram with the collected fluorescence signal with the channels filled with PBS before and after the immobilisation of the labelled antibodies on the surface of channel 4 and channel 9.

### The final integrated platform

**Fig. 7:**
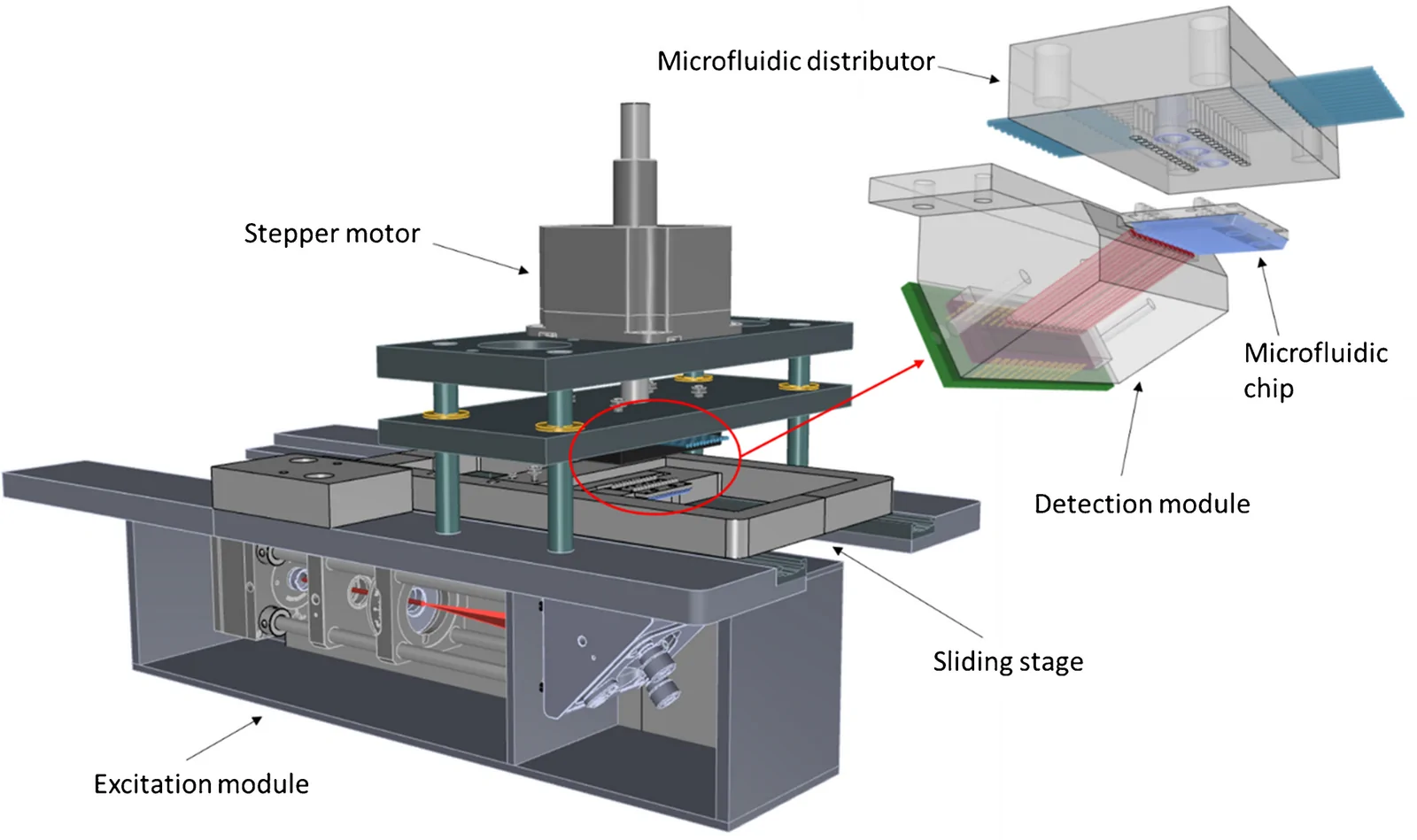
Rendering of the integrated optoelectronic and mechanical platform. The excitation module is shown in the lower section, while the microfluidic chip (highlighted by the red circle) is mounted on a sliding stage for precise alignment with the excitation pattern and detection module. The inset provides a detailed view of the chip-detector interface, including the WAF array, CCD sensor, and microfluidic distributor

The rendering of the optoelectronic and mechanical part of the final integrated platform is depicted in Fig. 7, highlighting the transition from individually optimised modules to a fully integrated POCT platform. In the lower part of Fig. 7, the excitation module previously described in the dedicated section is visible. The microfluidic chip, highlighted by the red circle, was mounted on a sliding stage that allows the chip to be inserted into the platform, aligning it with the diffracted laser lines and with the holder containing the WAF arrays and the camera. This configuration ensures consistent spatial alignment between excitation and detection, thereby reducing position-dependent variability in the measured fluorescence signal.

A stepper motor was used to precisely position and connect a custom-made stainless steel microfluidic distributor to the chip, ensuring reliable and reproducible fluidic interfacing through Teflon tubing and minimising operator-dependent variability during chip connection. The inset in the upper left provides a detailed view of the chip-detector interface, including the WAF array, CCD sensor, and microfluidic distributor. This integrated configuration improves mechanical stability and preserves optical coupling efficiency, thereby reducing signal fluctuations associated with misalignment.

This assembly constituted a stand-alone unit, which was then inserted into the enclosure of the final prototype. Figure S3 in the Supplementary Materials shows a photograph of the enclosure, where the pumps and microfluidic valves, the PC, the USB hub for data connections, and the power supply unit are visible.

A complete view of the platform, including the fluidic handling system, is reported in Figures S3 and S4 in the Supplementary Materials. The fluidic architecture comprised:

- two syringe pump modules (Hamilton PSD/4) with different valve heads (one six-port and one four-port configuration, each including a syringe port);
- three valve positioners (Hamilton MVP/4), including one four-port and two eight-port distribution valves located in proximity to the chip;
- a fluidic mixer implemented as spiral-shaped tubing.

This modular configuration enabled flexible routing and mixing of reagents required for multi-step immunoassays. In particular, the proximity of the valve system to the chip allowed precise control of reagent exchange within the microchannels, improving assay repeatability and reducing the risk of cross-contamination.

The fluidic components were arranged to minimise the total internal volume and reduce dead volumes, thereby limiting the priming volume of the fluorescence module and improving assay response time, which is particularly advantageous in point-of-care settings where rapid time-to-result and low reagent consumption are essential.

**Fig. 8:**
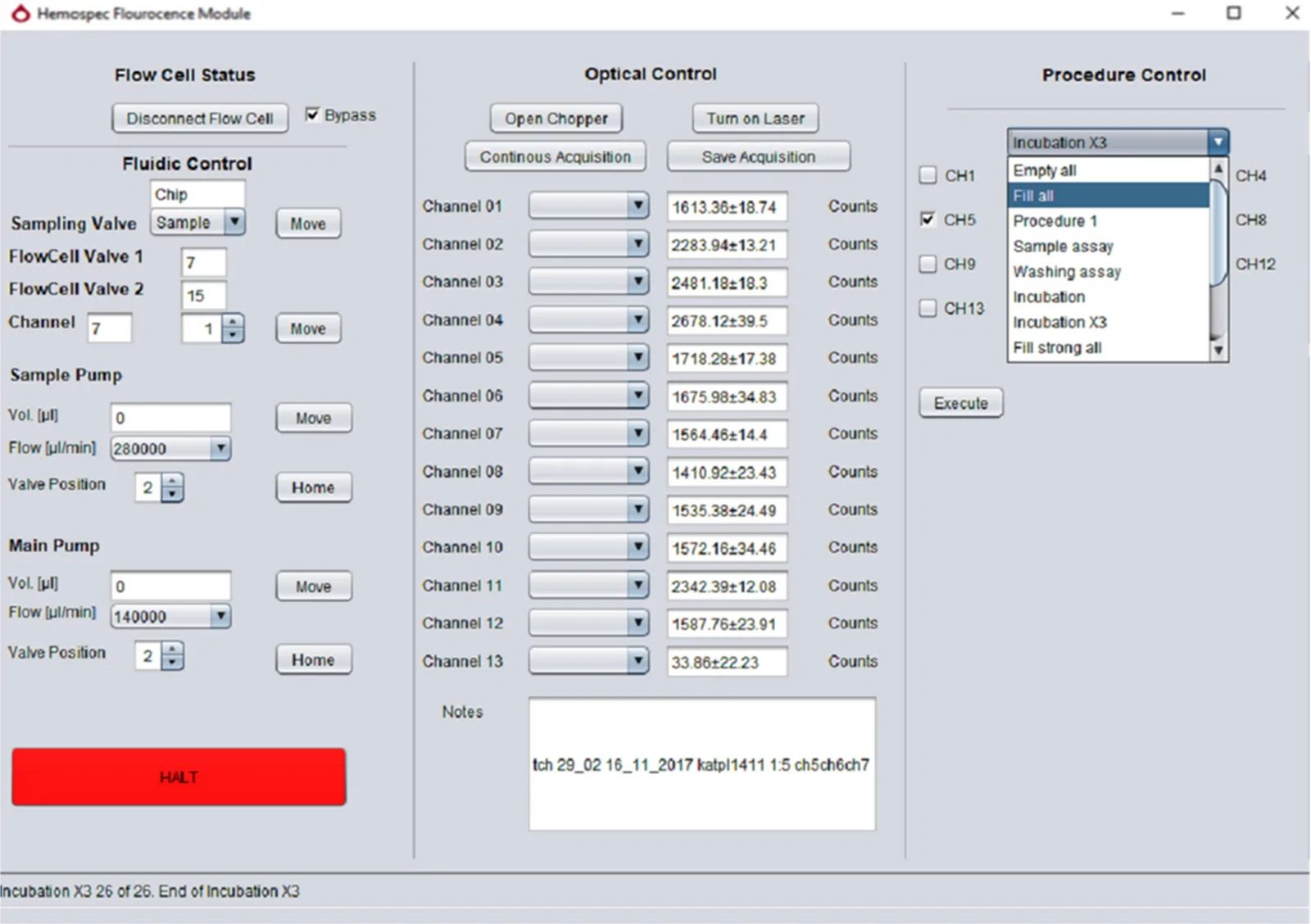
Graphical user interface (GUI) for system control. The software integrates fluidic handling and optical acquisition through four main sections: flow cell status, manual fluidic control, optical control and fluorescence acquisition, and automated protocol execution. This interface enables both stepwise operation and fully automated assay workflows

A small form factor PC with MS Windows operating system was located inside the final prototype box (Figure S3 in the Supplementary Materials) and externally connected through HDMI and USB ports. A dedicated graphical user interface (GUI), developed in Java, enabled integrated control of the optical and fluidic subsystems, allowing both manual operation and fully automated execution of assay protocols. In Fig. 8, the Control Window of the Fluorescence Module organised into four sections is presented:

- flow cell status (upper left): indicates and controls the fluidic connection of the flow cell
- fluidic control (lower left): enables manual operation of pumps and valves for stepwise assay development
- optical control and fluorescence acquisition (centre): allows the user to manage optical components and perform fluorescence measurements
- procedure control (right panel): provides access to pre-programmed automated assay protocols.

The integration of fluidic and optical control within a single software environment ensured proper synchronisation between reagent handling and fluorescence acquisition, thereby reducing timing-related errors and improving measurement reproducibility. Moreover, the availability of an integrated software interface enabling full control of both the fluidic and optical modules allowed the execution of fully automated measurement protocols. The availability of pre-programmed protocols supported the standardisation of assay procedures, which is a key requirement for reliable POCT deployment.

**Fig. 9:**
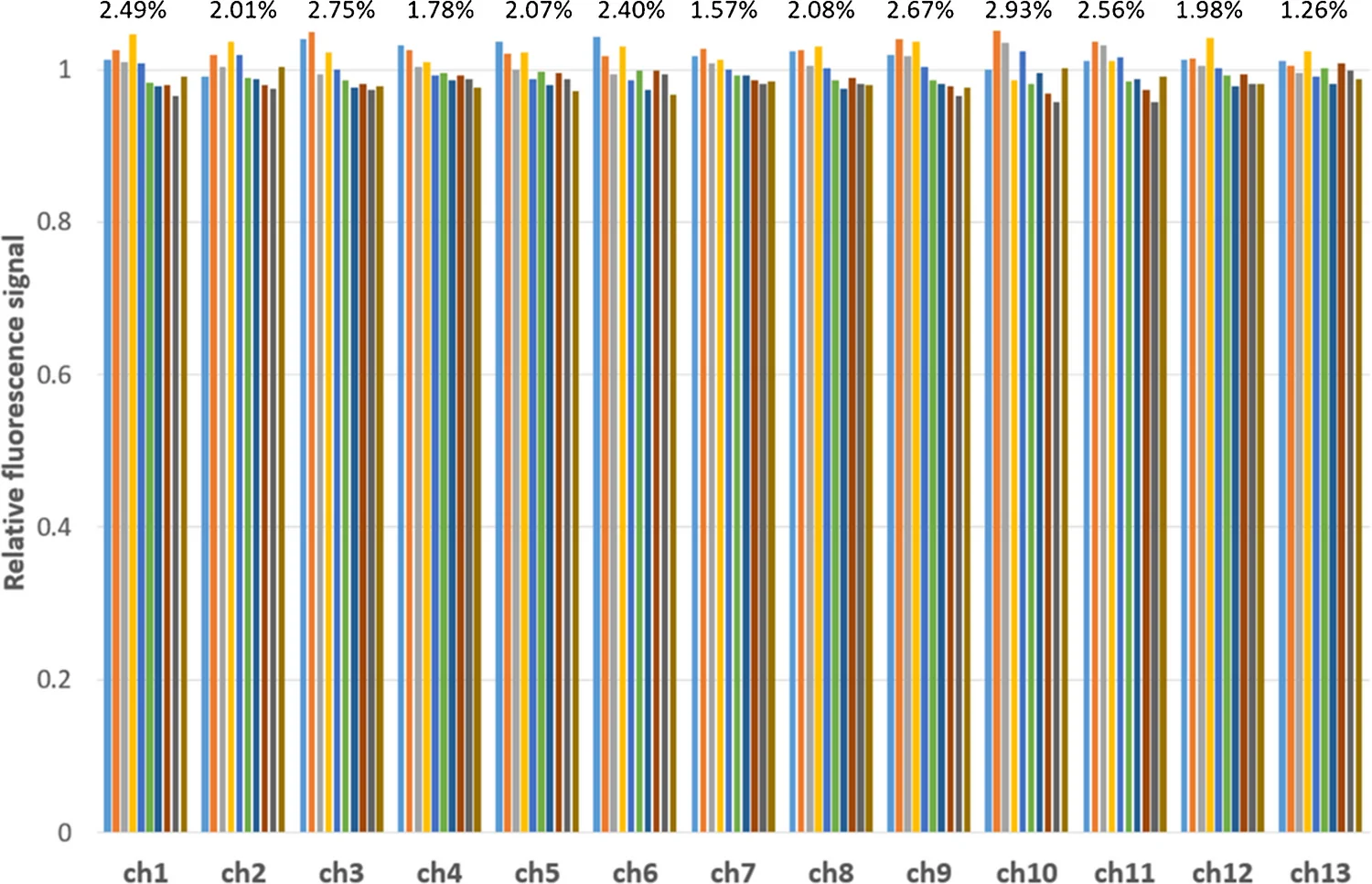
Assessment of system repeatability through repeated loading and unloading cycles (*n* = 10). For each channel, the fluorescence signal is normalised to the average value, and the corresponding coefficient of variation (CV) is reported

In this framework, the operational reliability was assessed by evaluating the stability and repeatability of the fluorescence signal upon repeated loading and unloading of the microfluidic chip with the sliding stage. For this purpose, the stability of the acquired optical signal as a function of repeated loading and unloading of the optical chip was evaluated. The chip microchannels, previously filled with anti-mouse IgG 5 µg/mL labelled with Alexa Fluor 647, were allowed to dry completely prior to each measurement in order to avoid any possible variation of the signal induced by leakage of the solution present in the channels during the loading-unloading procedure. The measurement of the emitted fluorescence was repeated ten times, and between each acquisition, the same chip was removed from the sliding stage and loaded again. The results are summarised in Fig. 9, where, for each channel, the acquired optical signal normalised to the average signal is reported with the indication of the CV value on the top of every channel. By inspecting the data reported in the histogram, a slight decrease in fluorescence signal can be observed for some channels when comparing the first insertions with the last ones. On average across the 13 channels, this corresponds to a small reduction in signal when comparing the first five measurements with the last five, with an overall variation of approximately 2%, while no systematic trend is present within each subset. This behaviour is attributed to a mechanical settling of the chip on the stage. For this reason, a short pre-conditioning (stabilisation) sequence is routinely performed when introducing a new chip, ensuring optimal measurement stability.

Overall, the low variability observed upon repeated loading/unloading cycles highlights the robustness of the platform in conditions compatible with routine POCT use. The temporal stability of the intensity distribution was further confirmed by these repeated measurements, which showed minimal variability despite sample removal and repositioning (see Fig. 9), making the platform suitable for rapid point-of-care testing applications where time-to-result is critical. On this basis, the platform was subsequently validated at the bioanalytical level by implementing a model fluorescence sandwich immunoassay targeting C-reactive protein (CRP) on the multichannel microfluidic chip.

**Fig. 10:**
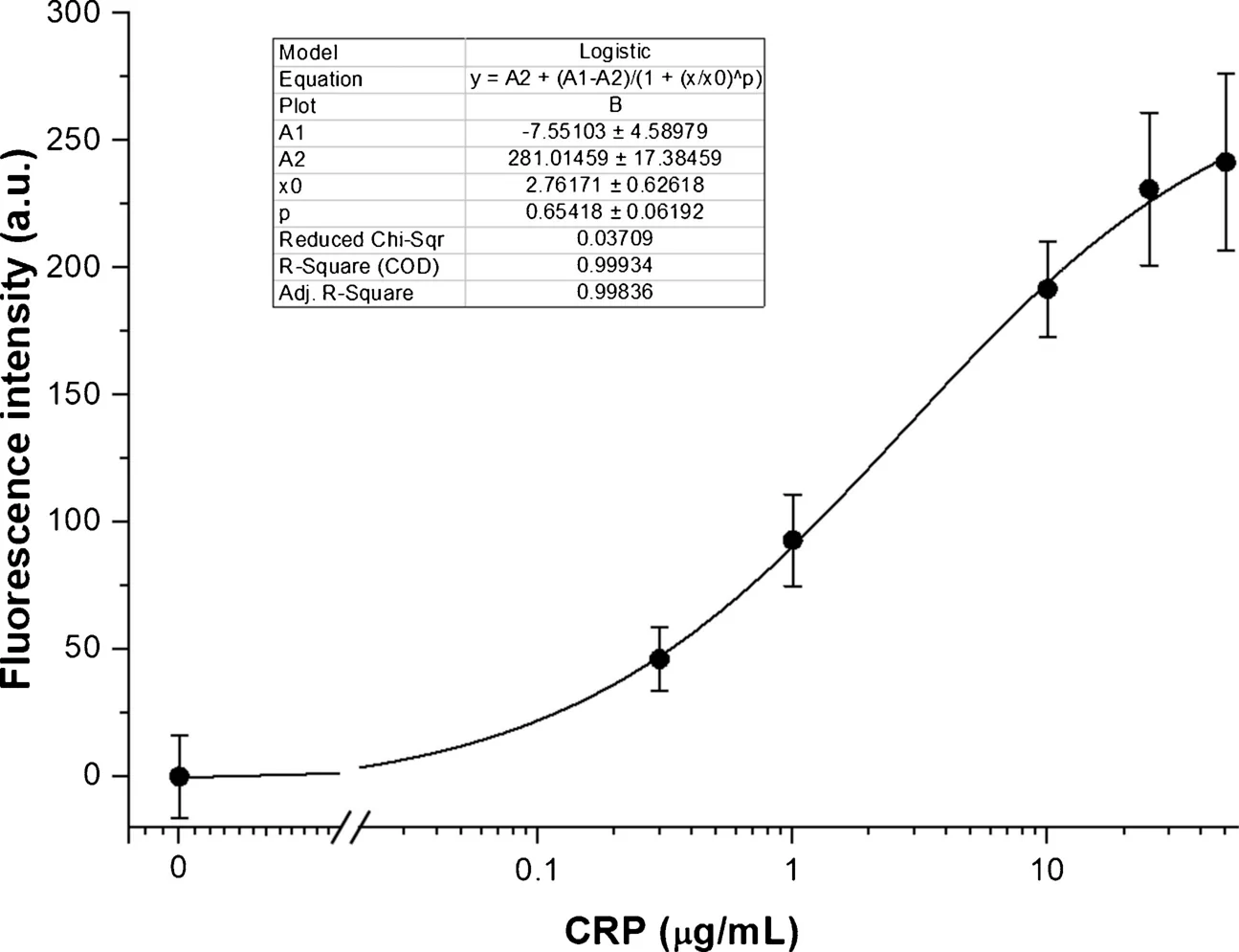
Calibration curve for CRP detection in plasma, obtained by spiking CRP into CRP-deficient plasma diluted 1:5 in PBS. Experimental data were fitted using a logistic model

The assay relied on the monoclonal anti-human CRP capture antibody (clone C5) and an Alexa Fluor 647-labelled detection antibody (clone C7). The assay was performed through the sequential flow protocol described in SI, with a total assay time of 30 min: in particular, steps 1–5 in Table S1 in the Supplementary Materials were used for the injection and 10-min incubation of CRP followed by washing and background acquisition. The procedure steps 6–8 consisted in the injection and 10-min incubation of the fluorescently labelled detection antibody, followed by the final signal acquisition. To evaluate assay applicability in plasma, a calibration was performed by spiking CRP into CRP-deficient plasma diluted 1:5 in PBS (Fig. 10). The baseline signal measured for CRP-deficient plasma (approximately 8 a.u.) was subtracted from all data; considering the intrinsic complexity of plasma as a biological matrix, this condition already provides a stringent assessment of non-specific effects, and the residual background remains significantly lower than the signal obtained at the lowest CRP concentration tested. The experimental data were fitted by using a logistic model, where the parameters A1 and A2 represent the lower and upper asymptotes, x₀ corresponds to the CRP concentration at 50% of the dynamic range, and p is related to the slope of the curve at x₀. From this calibration curve, an LOD of 0.32 µg/mL was calculated.

Intra‑chip variability was assessed by measuring multiple channels on the same chip and resulted in coefficient of variation (CV) values of 12% at a CRP concentration of 0.1 µg/mL (*n* = 8) and 10% at 10 µg/mL (*n* = 5). Inter-chip variability was evaluated across four different chips (11 microchannels in total) at a CRP concentration of 10 µg/mL with a CV of 15% confirming good reproducibility between chips. Based on the characterisation of the optical platform reported in the previous sections, the observed variability can be reasonably attributed predominantly to the immunoassay steps [35, 36], while contributions from the optical subsystem and potential inter-channel crosstalk are expected to be comparatively minor.

Two simulated plasma samples were produced by spiking CRP-deficient plasma with CRP 5 and 50 µg/mL and then diluting them 1:5 with PBS (final CRP concentrations 1 µg/mL and 10 µg/mL). Results are reported in Table 2.

**Table 2:** Results on simulated plasma samples with CRP concentrations of 1 μg/mL and 10 μg/mL after dilution 1:5 in PBS

CRP in simulated sample	Estimated CRP concentration (1:5 diluted sample)	Estimated CRP concentration (undiluted sample)	Recovery
5 µg/mL	1.05 µg/mL	5.25 µg/mL	105%
50 µg/mL	9.3 µg/mL	46.5 µg/mL	93%

The results presented for the CRP immunoassay demonstrate the capability of the proposed platform to perform quantitative fluorescence measurements in clinically relevant conditions, while maintaining good reproducibility and low inter-channel variability. Beyond the analytical performance obtained for this specific biomarker, the main strength of the proposed approach resides in the underlying optical and system architecture, which differs significantly from conventional fluorescence-based POCT platforms reported in the literature.

In contrast to widely adopted fluorescence-based POCT configurations relying on imaging-based detection or sequential interrogation of individual sensing regions [1, 5, 6, 14, 16, 24], the present system integrates parallel excitation and parallel signal collection within a fully static optical architecture. In many reported implementations, multichannel detection is achieved either through wide-field imaging or time-sequential readout schemes, which can introduce trade-offs in terms of acquisition time, optical complexity, or scalability. The approach proposed here enables truly parallel interrogation, where excitation and signal collection occur simultaneously across all microchannels without the need for scanning or imaging optics.

From this perspective, it is important to emphasise that the overall assay duration in the proposed platform is no longer limited by the optical readout, but rather by the biochemical kinetics of the specific assay. In the case of CRP detection, a total assay time of 30 min was required to ensure adequate binding and signal development. However, the decoupling between optical interrogation and assay chemistry represents a key advantage of the system, as it enables the modulation and optimisation of the total analysis time depending on the target analyte and assay format. Faster assays could be implemented for biomarkers with more rapid binding kinetics, while longer incubation protocols could be adopted when higher sensitivity is required, without any impact on the readout speed.

This flexibility, combined with the intrinsic fast optical response, is particularly relevant in the context of point-of-care testing, where time-to-result and adaptability to different clinical scenarios are critical. Moreover, the integration of the optical and fluidic subsystems within a single automated platform ensures reproducible assay execution and minimises user-dependent variability, further supporting the suitability of the platform for decentralised diagnostic applications.

Overall, the proposed approach demonstrates how the implementation of a parallel optical interrogation strategy, coupled with an optimised microfluidic handling system, can address key limitations of current fluorescence-based POCT devices, particularly those related to sequential readout and acquisition time scaling with the number of channels. The proposed architecture enables acquisition times on the order of seconds that are independent of the number of interrogated microchannels, while maintaining a compact and fully static optical configuration. In this framework, it is worth noting that several commercially available POCT CRP systems, such as QuikRead go, Afinion, and LumiraDx, rely on optical detection schemes and are optimised for specific clinical use scenarios; therefore, the analytical performance reported here, including a limit of detection of 0.32 µg/mL, should be interpreted as a proof of concept demonstration of the platform capability rather than a fully optimised assay, while already falling within a concentration range that is relevant for clinical decision-making [37]. While the total assay time of 30 min is longer than that of rapid lateral flow tests, it should be noted that the latter rely on simplified assay formats based on passive capillary flow and limited control over fluid dynamics [38], whereas the present approach is based on a fully controlled microfluidic immunoassay designed to ensure quantitative and reproducible measurements. In this context, the current results should be considered as a proof of concept of the performance of a fully integrated optoelectronic platform capable of rapid, stable, and parallel fluorescence readout within a controlled microfluidic framework by means of the measurement of a single analyte.

Despite these promising results, some aspects still require further optimisation. In particular, from the optical perspective, although the current configuration already ensures efficient parallel excitation and detection with low crosstalk, additional refinements aimed at further improving signal-to-noise ratio and channel isolation could be beneficial, particularly in the context of low-concentration biomarkers or highly multiplexed assays. Finally, while the current study focuses on single-analyte detection as a controlled validation of the platform, the extension towards multiplexed assays will require dedicated optimisation of bioassay design, surface functionalisation, and cross-reactivity control, in order to fully exploit the scalability potential of the multichannel architecture.

## Conclusions

In this work, a novel benchtop POCT platform based on parallel fluorescence interrogation of multichannel microfluidic chips has been developed and experimentally validated. The integration of a diffractive optical element for simultaneous excitation and a waveguide absorption filter array for parallel signal collection enabled the realisation of a fully static optical architecture, eliminating the need for mechanical scanning and significantly reducing the optical acquisition time to a few seconds.

The combination of this optical strategy with an optimised microfluidic system and an integrated software control environment resulted in a compact and automated platform capable of performing reproducible fluorescence measurements with low variability. The platform was successfully validated through the implementation of a CRP sandwich immunoassay, performed in plasma, demonstrating its capability to operate under clinically relevant conditions with satisfactory analytical performance.

A key outcome of this work is the decoupling between optical interrogation and assay kinetics, which allows the total analysis time to be dictated by the biochemical protocol rather than by the detection system. This feature provides a significant advantage for point-of-care (POCT) applications, as it enables flexible optimisation of assay duration depending on the specific biomarker and clinical requirements, while maintaining a fast readout.

Overall, the proposed approach addresses some of the major limitations of current fluorescence-based POCT devices, particularly in terms of acquisition speed, scalability, and system complexity. These results highlight the potential of parallel optical architectures for the development of next-generation diagnostic platforms capable of combining rapid response, multiplexing capability, and analytical reliability in a compact and robust format.

## Supplementary Information

Below is the link to the electronic supplementary material.

Supplementary file1 (PDF 775 KB)

## Data Availability

All data that are included in the paper are available from the authors by request.
