# Supplementary material for: Parallel fluorescence interrogation of multichannel chips for rapid point-of-care testing
Source: Anal Bioanal Chem. 2026 Aug 8;418(23):7937–51. doi: 10.1007/s00216-026-06719-4 (PMC13744510; doi:10.1007/s00216-026-06719-4)
Supplement: Supplementary file 1 [file 216_2026_6719_MOESM1_ESM.pdf]

Supplementary materials

Parallel fluorescence interrogation of multichannel chips for rapid point of care testing

Cosimo Trono[1†], Simone Berneschi[1†], Sara Tombelli[1†], Ambra Giannetti[1], Barbara Adinolfi[1], Francesco Chiavaioli[1], Giampiero Porro[2], Luca Bolzoni[2], Giovanna Quarto[2], Romeo Bernini[3], Gianluca Persichetti[3], Genni Testa[3], Francesco Baldini[1*]

1) *Istituto di Fisica Applicata "Nello Carrara", Consiglio Nazionale delle Ricerche, Via Madonna del Piano 10, 50019 Sesto Fiorentino, Italy*
2) *Datamed Srl, Via Amerigo Vespucci, 2/4/6, Peschiera Borromeo (Milan), Italy*
3) *Istituto per il Rilevamento Elettromagnetico dell'Ambiente, Consiglio Nazionale delle Ricerche, Via Diocleziano, 328, 80124 Napoli, Italy*

[†]These authors contributed equally to the work

[*]Corresponding author email: f.baldini@ifac.cnr.it

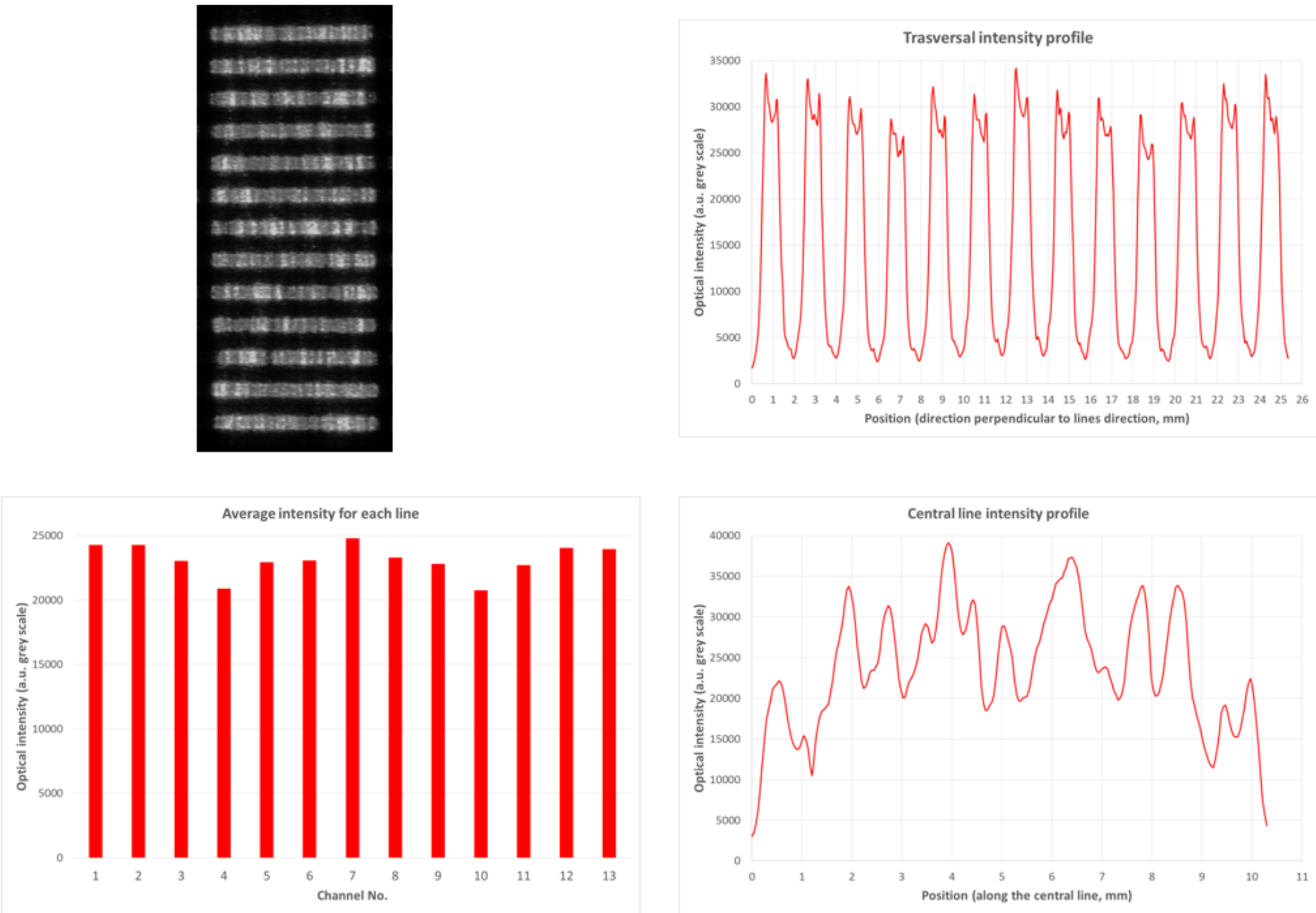

Figure S1. Assessment of the diffraction pattern quality in the case of LD spot long axis perpendicular to the diffracted lines: (a) photograph of the diffraction pattern projected onto a screen, (b) transverse intensity profile, (c) average intensity for each line, and (d) longitudinal intensity profile of the central line.

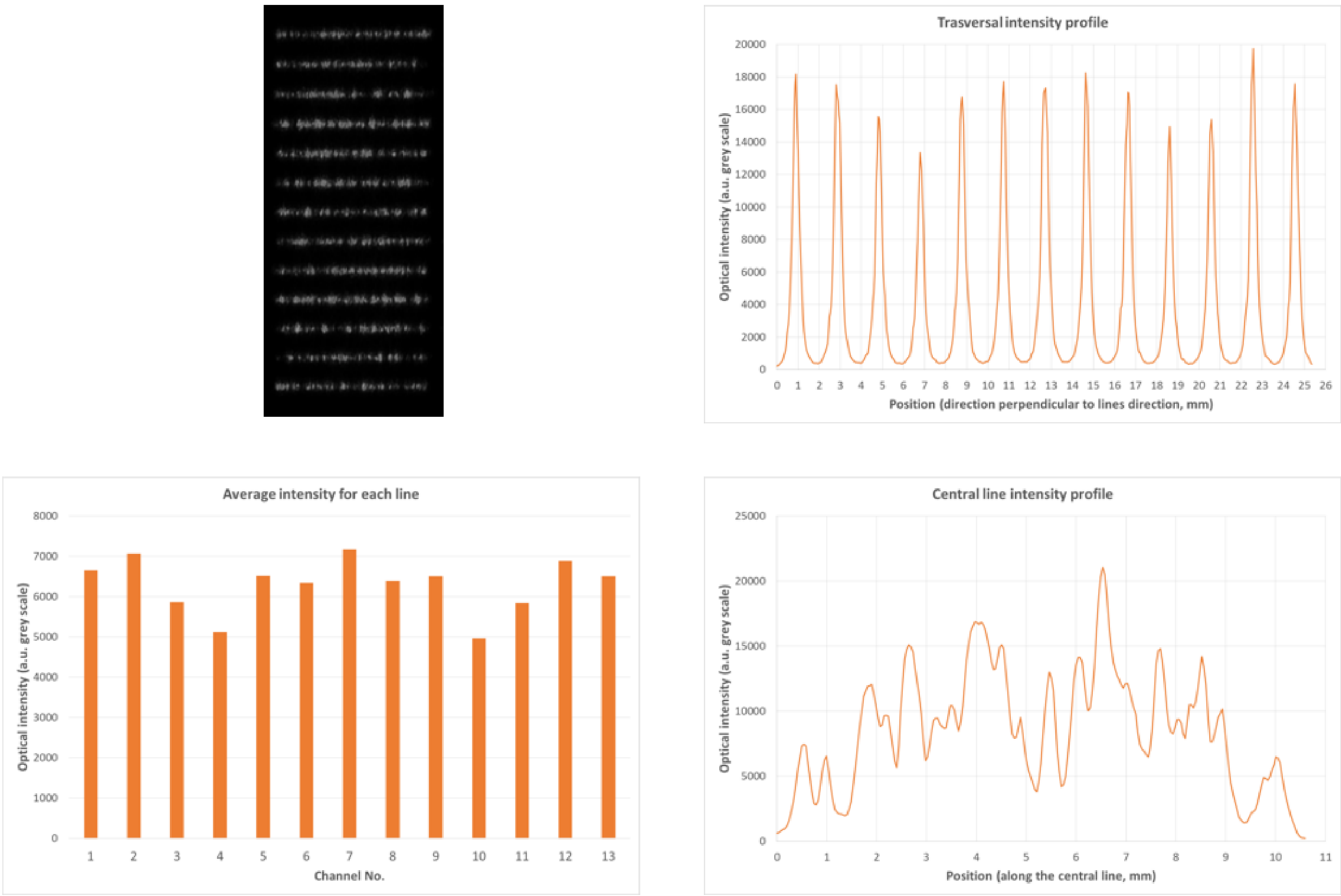

Figure S2. Assessment of the diffraction pattern quality in the case of Circular LD spot: (a) photograph of the diffraction pattern projected onto a screen, (b) transverse intensity profile, (c) average intensity for each line, and (d) longitudinal intensity profile of the central line.

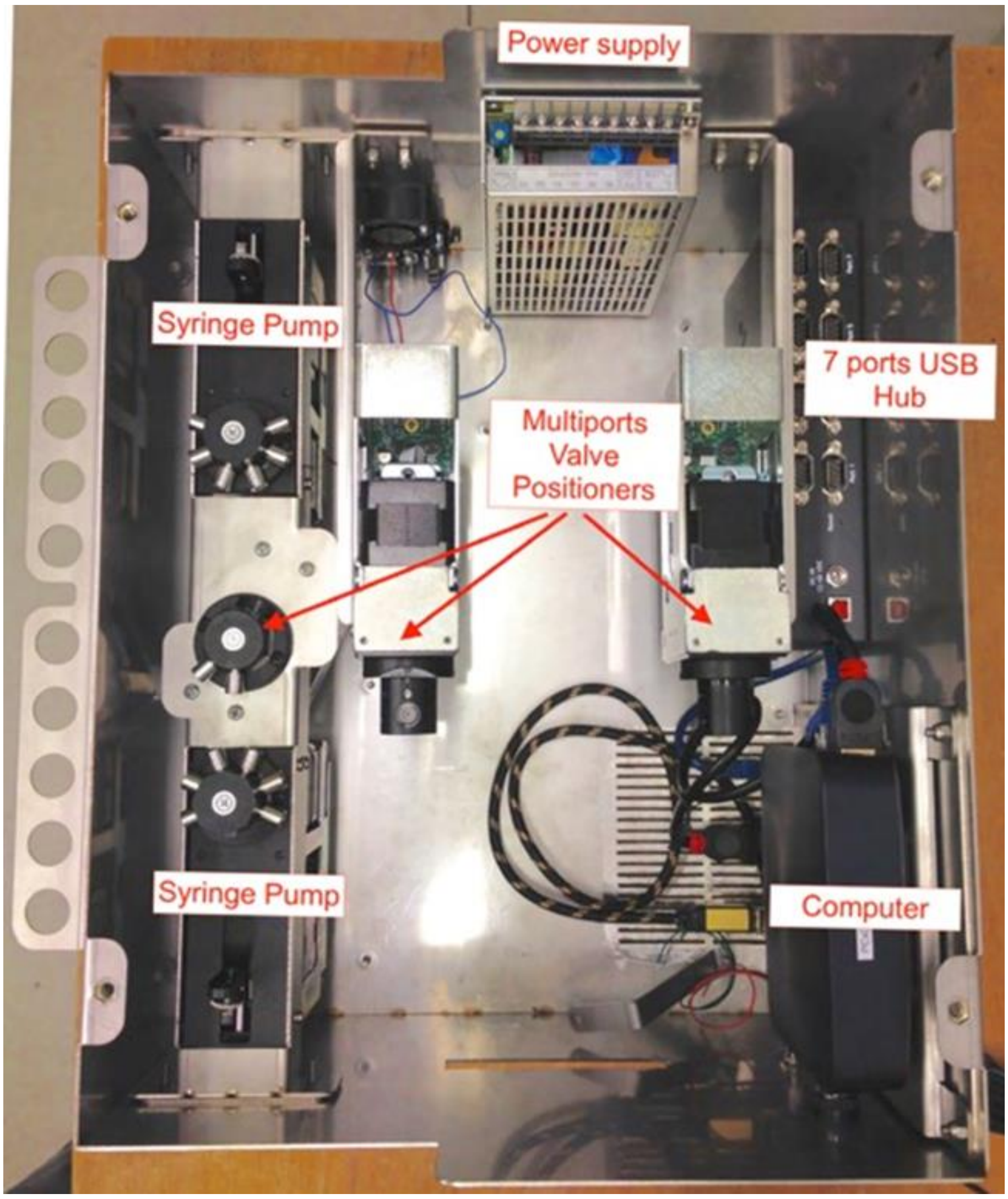

Figure S3. Photograph of the enclosure of the final prototype, showing the pumps and microfluidic valves, the PC, the USB hub for data connections, and the power supply unit.

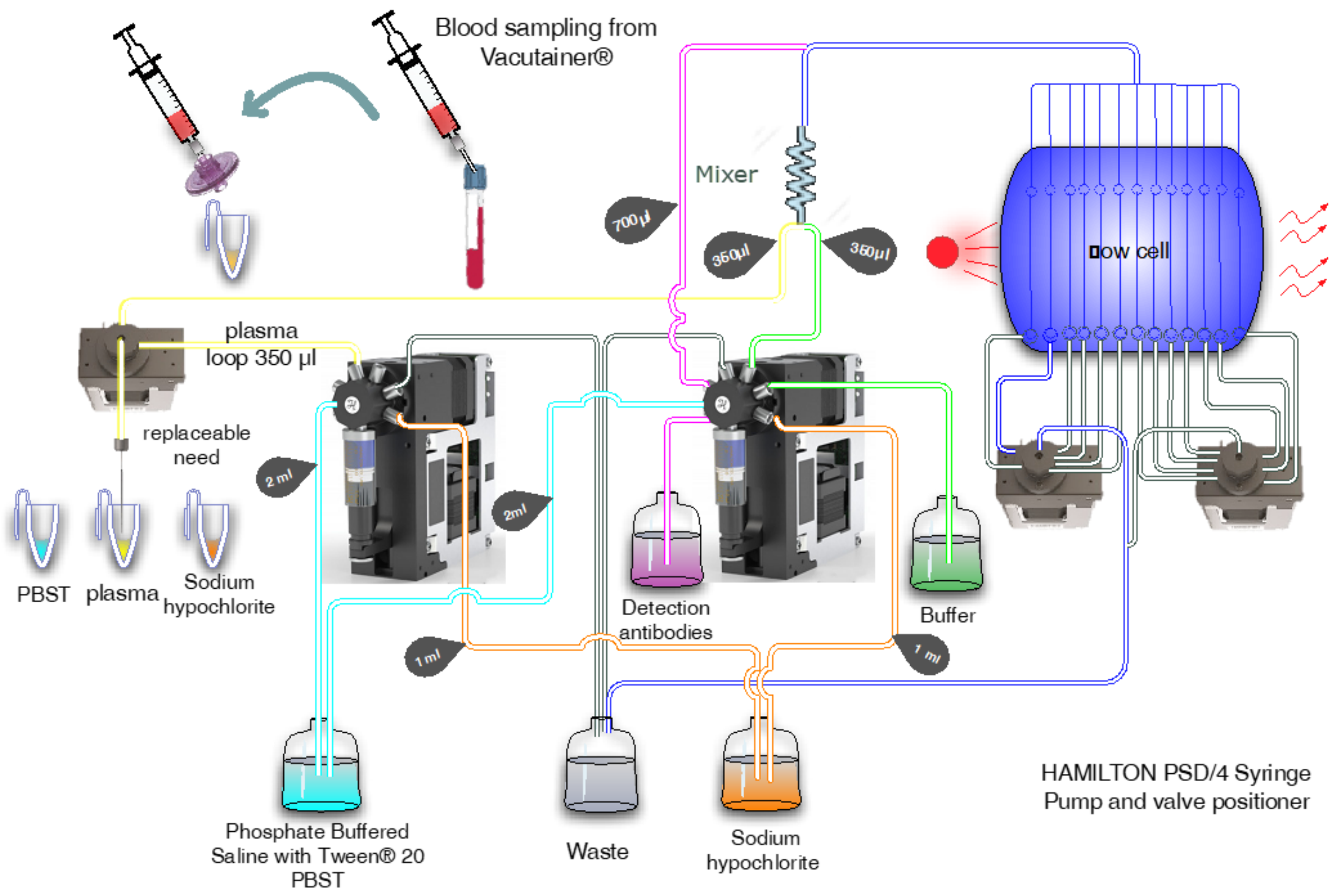

Figure S4. Schematic of the fluidic handling system, comprising two syringe pump modules equipped with valve heads, three valve positioners and a fluidic mixer implemented as spiral-shaped tubing.

Measurement procedure with the final integrated device

The list of fluidic procedures reported in Table S1 has been established for the measurements into the integrated device.

1. FILL PBST (select channels);<br>2. SAMPLE ASSAY (select channels and volume (µL); place sample tube in XXX vial);<br>3. INCUBATION (select channels);<br>4. WASHING ASSAY (select channels);<br>5. WASHING LOOP SOFT;
SAVE ACQUISITION (name of the file: XXX_bkg)
6. SAMPLE ASSAY (select channels and volume (µL); place sample tube in Ab* vial);<br>7. INCUBATION (select channels);<br>8. WASHING ASSAY (select channels);
SAVE ACQUISITION (name of the file: XXX_meas)

Table S1. List of fluidic procedures established for the measurements on the final integrated device.

A description of the different procedures is given below:

-FILL PBST: this procedure fills the selected channels with PBST, which is the chosen running and washing buffer;

-SAMPLE ASSAY: this procedure is homogeneously distributing the solution/sample to be tested among the selected channels;

-INCUBATION: this procedure is repeatedly moving the injected solution/samples into the channels by pushing 15 µL and pulling 10 µL with short intermediate and final incubation steps;

-WASHING ASSAY: this procedure is washing the selected channels with PBST at 250 µL/min;

-WASHING LOOP SOFT: this procedure is first filling the loop with PBST by the sample tube and then is emptying the loop by intake of air from the same tube;

-SAVE ACQUISITION: this step, which is not fluidic, is used to register the signal after the incubation with the solution/sample to be tested, which is used as signal background, and after the incubation with the labelled detection antibody. The measurement is actuated by the command Save Acquisition on the module interface.
